# HEALTH SEEKING BEHAVIOR FOR YOUNG ONSET DEMENTIA IN WESTERN COUNTRIES AND EAST ASIA: A SCOPING REVIEW

**DOI:** 10.1093/geroni/igae098.3994

**Published:** 2024-12-31

**Authors:** Ko-Xin Chen, Han-Yun Chang, Valerie Cotter

**Affiliations:** National Cheng Kung University, Tainan, Taiwan (Republic of China); Johns Hopkins University School of Nursing, Baltimore, Maryland, United States; Johns Hopkins University School of Nursing, Baltimore, Maryland, United States

## Abstract

**Aim:**

We aimed to investigate the impact of culture in Western countries and East Asia on the health-seeking behavior in young onset dementia.

**Background:**

While previous research has largely focused on the impact of culture on health-seeking behaviors in late onset dementia, a gap exists in understanding these influences in young onset dementia since they present unique challenges and needs.

**Design and Methods:**

A scoping review was conducted using Arksey and O’Malley’s framework. Databases searched included Embase, Ovid MEDLINE, Cochrane Trials, CINAHL, PsycINFO, Scopus, Airiti Library, and Taiwan’s National Digital Library of Theses and Dissertations, with coverage up to July 22, 2024. The PRISMA-ScR checklist was employed to ensure the rigor of this scoping review.

**Results:**

From 1,611 retrieved articles 27 studies were included. The synthesized results were analyzed using the Health Belief Model. Two main themes emerged from the analysis: (i) perceived susceptibility, severity, and barriers, with subthemes including stigma, financial impact of working, psychological and emotional effects, diagnostic challenges, and access to healthcare; and (ii) perceived benefits, self-efficacy, and cues to action, with subthemes including welfare, individualistic or collective values, meaningful activities, and environmental support.

**Conclusion:**

Health-seeking behavior for young onset dementia is influenced by cultural differences between Western countries and East Asia. Specifically, Western cultures emphasize individualistic values, while East Asian cultures prioritize collective decision-making. Clinical implication: It is crucial to develop informed and culturally appropriate care for persons with young onset dementia that is tailored to the distinct needs of Western and East Asian populations.

